# Deletion or insertion in the first immunoglobulin-plexin-transcription (IPT) domain differentially regulates expression and tumorigenic activities of RON receptor Tyrosine Kinase

**DOI:** 10.1186/1476-4598-9-307

**Published:** 2010-11-29

**Authors:** Qi Ma, Kun Zhang, Sunny Guin, Yong-Qing Zhou, Ming-Hai Wang

**Affiliations:** 1 Laboratory of Cancer Biology in State Key Laboratory for Diagnosis and Treatment of Infectious Diseases, First Affiliated Hospital, Zhejiang University School of Medicine, Hangzhou, P. R. China 310003; 2 Cancer Biology Center and Department of Biomedical Sciences, Texas Tech University Health Sciences Center School of Pharmacy, Amarillo, TX 79106, USA; 3 Division of Neurosurgery, First Affiliated Hospital, Zhejiang University School of Medicine, Hangzhou, P. R. China 310003

## Abstract

**Background:**

Activation of the RON receptor tyrosine kinase, a member of the c-MET family, regulates tumorigenic phenotypes. The RON extracellular domains are critical in regulating these activities. The objective of this study was to determine the role of the first IPT domain in regulating RON-mediated tumorigenic activities and the underlying mechanisms.

**Results:**

Two RON variants, RON160 and RON^E5/6in ^with deletion and insertion in the first IPT domain, respectively, were molecularly cloned. RON160 was a splicing variant generated by deletion of 109 amino acids encoded by exons 5 and 6. In contrast, RON^E5/6in ^was derived from a transcript with an insertion of 20 amino acids between exons 5 and 6. Both RON160 and RON^E5/6in ^were proteolytically matured into two-chain receptor and expressed on the cell surface. RON160 was constitutively active with tyrosine phosphorylation. However, activation of RON^E5/6in ^required ligand stimulation. Deletion resulted in the resistance of RON160 to proteolytic digestion by cell associated trypsin-like enzymes. RON160 also resisted anti-RON antibody-induced receptor internalization. These features contributed to sustained intracellular signaling cascades. On the other hand, RON^E5/6in ^was highly susceptible to protease digestion, which led to formation of a truncated variant known as RONp110. RON^E5/6in ^also underwent rapid internalization upon anti-RON antibody treatment, which led to signaling attenuation. Although ligand-induced activation of RON^E5/6in ^partially caused epithelial to mesenchymal transition (EMT), it was RON160 that showed cell-transforming activities in cell focus formation and anchorage-independent growth. RON160-mediated EMT is also associated with increased motile/invasive activity.

**Conclusions:**

Alterations in the first IPT domain in extracellular region differentially regulate RON mediated tumorigenic activities. Deletion of the first IPT results in formation of oncogenic variant RON160. Enhanced degradation and internalization with attenuated signaling cascades could be the mechanisms underlying non-tumorigenic features of RON^E5/6in^.

## Background

The RON (recepteur d'origine nantais) receptor tyrosine kinase belongs to the MET proto-oncogene family [1,2], which plays a critical role in epithelial cell homeostasis and tumorigenic development [3]. Expression of RON has been found mainly in cells of epithelial origin although certain tissue macrophages and immune cells also express the RON mRNA and protein [4-6]. Accumulated evidences have indicated that aberrant RON expression, characterized by protein overexpression and generation of various variants, contributes to pathogenesis of epithelial cancers [7,8]. Immunohistochemical staining has demonstrated that RON is overexpressed in more than 40% of primary cancer samples from breast, colon, and pancreatic tissues [4,9-11]. Increased RON expression has also been considered as a validated prognostic factor for predicting disease progression and survival rate in certain cancer patients [10,12]. Although RON gene mutations were not found in primary cancer samples, aberrant splicing resulting in formation of various tumorigenic RON variants is frequently observed in primary colon, breast, and brain tumors [7,13,14]. Functional analysis has revealed that RON activation promotes malignant phenotype of cancer cells [3]. In tumor cells overexpressing RON, cells undergo epithelial to mesenchymal transition (EMT) featured by spindle-like morphology, diminished E-cadherin expression, and increased vimentin expression [15,16]. EMT is a unique phenotype observed in cancer stem cells and is a critical process required for cancer metastasis [17]. Evidence has also indicated that altered RON expression results in increased survival and pro-apoptotic activity of tumor cells [18,19]. These activities of RON help to sustain tumor growth under hostile environment such as hypoxia [3,19,20]. Recent studies further demonstrate that abnormality in RON expression contributes to acquired resistance of cancer cells to conventional chemotherapeutics [21]. We have recently observed that down-regulation of RON expression under chronic hypoxia is a mechanism contributing to the insensitivity of tumor cells towards small molecule inhibitor-induced inhibitory or cytotoxic activities [22]. Clearly, aberrant RON expression is a pathogenic factor contributing to cancer development and malignant progression. Such abnormality also provides the molecular basis of targeting RON for potential therapeutic intervention [23].

As described above, aberrant RON expression is featured by generation of biologically active RON variants [7,13,14]. Currently, seven RON variants including RON170, RON165, RON160, RON155, RONp110, RON85, and RON52 have been identified in primary cancer samples and in established cell lines [7,14,24]. One of the tumorigenic variants is RON160, which is constitutively active and has oncogenic activities *in vivo *[13]. RON160 is produced by a RON mRNA transcript through alternative splicing that eliminates 109 amino acids in the RON extracellular domain [13]. These amino acids are encoded by exons 5 and 6, which constitute the first IPT domain in the RON β-chain [25]. The β-chain extracellular sequences harbor a cluster of four IPT units between sema and transmembrane segment [25-27]. The first IPT unit contains 103 amino acids (from Pro^569 ^to Asp^671^) and is featured by immunoglobulin-like fold [25]. The functions of the second and third IPT units are currently unknown. The fourth IPT unit is critically important in regulating RON protein maturation and cell surface expression [28,29]. Currently, the mechanism of how the deletion of the first IPT domain resulting in oncogenic conversion is largely unknown. It is reasoned that the deletion causes RON conformational change and leads to spontaneous dimerization, which causes constitutive receptor phosphorylation and increased intracellular signaling activation [13].

The purpose of the present work is to determine the role of the first IPT unit in the RON extracellular sequences in regulating RON-mediated tumorigenic activities in epithelial cells. By studying two RON variants formed either by deletion of 109 amino acids coded by exons 5 and 6 or by insertion of 20 amino acids between exons 5 and 6, we observed striking differences in biochemical and biological properties. Clearly, deletion or insertion induced alterations in the first IPT domain have different biological consequences, which may have pathogenic implications in regulating RON-mediated activities.

## Materials and methods

### Cell Lines and Reagents

Human colon (HT-29, SW620, and SW837), breast (HCC-1937, MDA-MB231, T-47D, ZR-751, and MCF-7), and pancreatic (BxPc-3, L3.6pl, and Panc-1) cancer cell lines and NIH3T3 cells were from ATCC (Manassas, VA). Madin Darby canine kidney (MDCK) cells stably expressing RON or RON160 (M-RON or M-RON160 cells) were established as previously described [15]. Human MSP was provided by Dr. E. J. Leonard (National Cancer Institute, Bethesda, MD). Mouse monoclonal antibodies (mAb) specific to the RON extracellular sequences (clones Zt/g4 and Zt/c1) were used as preciously described [30]. Rabbit IgG antibody specific to RON C-terminal peptide was described previously [31]. Recombinant human furin was from New England BioLabs (Ipswich, MA). PD98059 (PD), SB203580 (SB) and wortmannin (WT) were from Calbiochem (San Diego, CA). Mouse mAb specific to phospho-tyrosine (PY-100), phospho-Erk1/2, AKT, and other signaling proteins were from Cell Signaling (Danvers, MA). Rabbit or goat IgG antibodies specific to E-cadherin, vimentin, or β-actin were from BD Transduction Laboratories (Lexington, KY).

### Reverse Transcription (RT)-Polymerase chain reaction (PCR) and DNA sequencing

RT-PCR was performed as previously described [32]. Briefly, total RNA was isolated from individual cell lines using Trizol (Invitrogen, CA). RT was carried out using 2 μg of total RNA with a SuperScript Preamplification kit (Invitrogen). PCR was conducted by using a pair of oligomers to amplify RON160 or RON^E5/6in ^cDNA fragments [32]. Amplified cDNA fragments were subcloned into the pGEM-T-easy vector (Promega) and sequenced at the Texas Tech University DNA Sequence Core facility (Lubbock, TX).

### Construction of the full-length RON^E5/6in ^cDNA and its expression in MDCK cells

The complete RON^E5/6in ^cDNA was constructed by replacing a fragment in the wild-type RON cDNA with an amplified 0.6 Kb fragment to create the full-length RON^E5/6in ^cDNA as previously described [32]. Transfection of MDCK cells with RON^E5/6in^, selection of stable cell lines, and Western blot analysis of protein expression were conducted as previously described [13].

### Immunoprecipitation and Western blot analysis

These methods were performed as detailed previously [31,32]. Cellular proteins (250 μg/sample) were used for immunoprecipitation by Zt/g4 (2 μg/sample) coupled to protein G Sepharose beads. Individual proteins were detected using specific antibodies in Western blot analysis under reduced conditions. Membranes were also reprobed with rabbit IgG antibody to β-actin to ensure equal sample loading [31,32].

### Immunofluorescent cell surface analysis

Fluorescent cell surface analysis was carried out as previously described [33]. Briefly, M-RON, M-RON160 or M-RON^E5/6in ^cells (1 × 10^6 ^cells/ml) were incubated with Zt/g4 (1 μg/sample/ml) followed by goat anti-mouse IgG coupled with FITC. Fluorescent intensity was determined by FACscan (Becton Dickinson) analysis as previously described [33]. In all assays, normal mouse IgG was used as the negative control.

### Protein micro-sequencing

M-RON and M-RON^E5/6in ^cells (3 × 10^6 ^cells/ml) in DMEM were treated with 0.05% of trypsin for various times. Cellular proteins (350 μg/sample) from lysates of M-RON or M-RON^E5/6in ^cells were first immunoprecipitated by Zt/g4 (1 μg/sample) coupled with protein G Sepharose beads [31]. Samples were then separated in 8% SDS-PAGE under reduced conditions followed by transfer to a poly(vinylidene difluoride) membrane (Problott; Applied Biosystems) [34]. The protein bands were identified, marked and analyzed directly on an Applied Biosystems 473A protein sequencer fitted with a reaction cartridge specifically designed for poly(vinylidene difluoride) bound samples at the Colorado State University Core facility (Ford Collin, Co).

### Cell migration assays

Wound healing assays were used to determine the ability of cells to migrate to cover the open space as previously described [24]. Cells were stimulated with MSP (2 nM) for 16 h. The percentage of open spaces covered by migrated cells was determined as previously described [24].

### Bioassays for cell focus formation and anchorage-independent growth in soft agar

Both assays were performed as previously described [13]. For focus formation, cultured NIH-3T3 cells in 30 mm diameter dish were transiently transfected with the pcDNA3.1 expression vector containing RON, RON160, or RON^E5/6in ^cDNA, respectively. Foci were counted after cells were maintained in DMEM with 1% FBS for 18 days. For colony formation, cells (2000 cells/dish) in 2 ml DMEM with 5% FBS and 0.3% agarose were seeded in a 30 mm diameter culture dish containing 0.7% agarose. The colony numbers were determined 18 days after initiation of cell culture.

## Results

### Different RON mRNA transcripts with alterations in the first IPT unit are present in colon, breast, and pancreatic cancer cells

**Figure 1:**
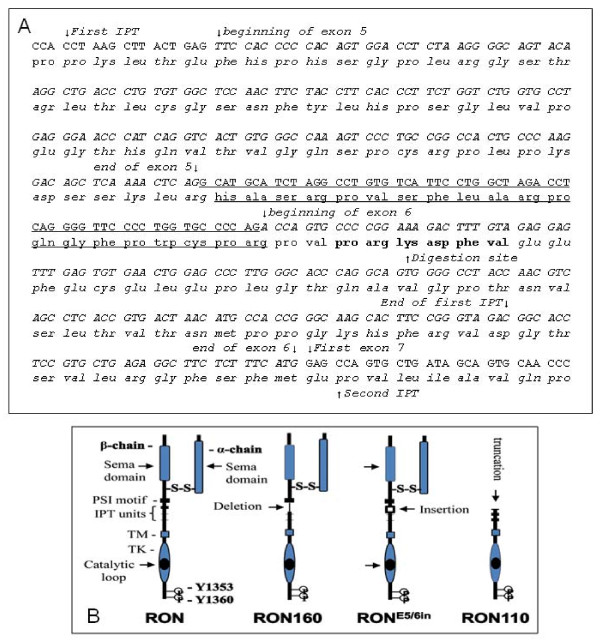
**Identification and cloning of RON mRNA transcripts with alterations in the first IPT domain in cancer cell lines**. **A**) Partial sequences of amplified RON cDNA fragments. The sequences show a deletion of 327 nucleotides coded by exons 5 and 6 and an insertion of 60 nucleotides between exons 5 and 6. The deleted sequences that encode 109 amino acids by exons 5 and 6 are italicized (as detected in RON160 cDNA). The inserted sequences encoding 20 new amino acids are underlined (as detected in RON^E5/6in ^cDNA). The beginning and ending of the first IPT unit are indicated. The first nucleotides of exons 5, 6, and 7 are marked with an arrow. The amino acids that act as the digestive site for trypsin-like serine proteases, which led to generation of RONp110, are shown in bold. **B**) Schematic representation of wild-type RON, RON160, RON^E5/6in^, and RONp110. Mature RON contains a sema domain (localized in both α and β-chains) followed by a PSI motif and four IPT units. The deletion of exons 5 and 6 and the insertion between exons 5 and 6 in the first IPT unit are indicated with arrows. Cleavage by trypsin-like serine protease in the digestive site results in a truncated variant known as RONp110. Two tyrosine residues (Y1353 and Y1360) in the C-terminal tail are indicated. TM: transmembrane domain; TK: tyrosine kinase domain.

Previous studies have shown that deletion of the first IPT unit coded by exons 5 and 6 results in formation of oncogenic variant RON160 [13]. To determine if other types of alterations exists in the first IPT unit, total RNA isolated from a panel of twelve cancer cell lines was subjected to RT-PCR analysis. The cDNA fragments were amplified by using primers that cover the first IPT unit and its surrounding sequences (^+^1646 to ^+^2184 from exons 4 to 7). Results in Table 1 and Figure 1A are the summary of the RT-PCR analysis. Three cDNA fragments, Fgm-I (0.54 kb), Fgm-II (0.21 kb), and Fgm-III (0.6 kb) were obtained (data not shown). The cDNA sequence analysis indicated that Fgm-I encodes a portion of wild-type RON, which was amplified in all eleven cell lines known to express RON. MCF-7 cells do not express RON [32] and were used as a negative control. Fgm-II showed a deletion of 109 amino acids coded by exons 5 and 6 and was observed in HT-29, SW620, SW837 and Du4475 cell lines. Expression of this transcript was consistent with previous studies showing the existence of RON160 in colon and other cancer cell lines [13].

**Table 1:** Identification of RON mRNA transcripts with alterations in the first IPT Unit in colon, breast, and pancreatic cancer cells.

		Types of RON mRNA transcripts*		
		
Cancer Cell lines	Tissue sources	Wild type (0.54 kb)	Deletion of exons 5 & 6 (0.21 kb)	Insertion btw exons 5 & 6 (0.6 kb)
**HT-29**	Colon	**+**	**+**	**+**
**SW620**	Colon	**+**	**+**	**+**
**HCT116**	Colon	**+**	-	-
**SW837**	Colon	**+**	**+**	-
**T-47D**	Breast	**+**	-	-
**Du4475**	Breast	**+**	**+**	**+**
**HCC1937**	Breast	**+**	-	-
**ZR-751**	Breast	**+**	-	-
**MDA-MB231**	Breast	**+**	-	-
**BxPC-3**	Pancreatic	**+**	-	-
**L3.6pl**	Pancreatic	**+**	-	-
**Panc-1**	Pancreatic	**+**	-	**+**
**MCF-7**	Breast	-	-	-

*The mRNA isolation and RT-PCR analysis were performed as detailed in Materials and Methods. Cell lines expressing mRNA transcripts of RON and its variants are marked as +. Deletion and insertion were determined by comparison with published RON gene sequences [25].

An interesting finding was the detection of a 0.6 kb Fgm III from HT-29, SW620, Du4475, and Panc-1 cells. Sequence analysis showed an insertion of 20-amino acids (60 nucleotides) between the last amino acid of exon 5 (Arg^627^) and the first amino acid of exon 6 (Pro^628^) (Figure 1A and Table 1). The inserted sequences were identical among fragments amplified from four cell lines. By comparing the genomic sequence of the RON gene [25], it was determined that 60 nucleotides belong to the intron sequence between exons 5 and 6, which were retained during the splicing process. The resulting product is a RON mRNA transcript, which should be expressed as a novel RON variant with insertion in the first IPT unit (designated as RON^E5/6in^). Thus, three specific mRNA transcripts encoding wild-type RON, RON160, and RON^E5/6in ^were amplified from several cancer cell lines. Schematic representations of RON160 and RON^E5/6in ^with deletion or insertion in the first IPT unit are shown in Figure 1B. Clearly, RON^E5/6in ^is a novel variant that has not been previously reported.

Although wild-type RON and RON160 were detected by Western blot analysis using rabbit IgG specific to the RON C-terminus, we were unable to distinguish wild-type RON and RON^E5/6in ^due to small differences in their protein size (data not shown). Moreover, since the molecular mass of RON^E5/6in ^is almost identical to that of wild-type RON, we were unable to confirm if the RON^E5/6in ^protein is expressed in RT-PCR positive cell lines. Nevertheless, existence of mRNA transcripts for RON160 and RON^E5/6in ^provides us an opportunity to study the significance of the first IPT alterations in regulating RON-mediated activities.

### RON160 and RON^E5/6in ^are both expressed on cell surface but showed different phosphorylation status

**Figure 2:**
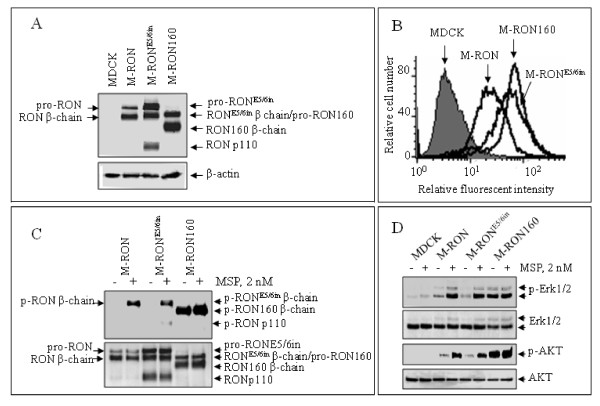
**Expression, localization, and phosphorylation of RON160 and RON**^**E5/6in **^**in MDCK cells**. **A**) Expression of RON160 and RON^E5/6in ^in MDCK cells. Cell lysates (50 μg/sample) from M-RON, M-RON160, or M-RON^E5/6in ^after 72 h incubation were analyzed by Western blot analysis using rabbit IgG antibody to RON C-terminus [29]. β-actin was used as the loading control. Both pro- and mature proteins were observed. The presence of RONp110 in M-RON^E5/6in ^cells was indicated. **B**) Immunofluorescent analysis of RON, RON160, and RON^E5/6in ^expression on cell surface. Cells (1 × 10^6 ^cells per sample) were incubated with 1 μg/ml of Zt/g4 specific to the RON extracellular domain [30]. Goat-anti-mouse IgG coupled with FITC was used as the secondary antibody. MDCK cells were used as the negative control. Fluorescent intensity of individual samples was analyzed by FACScan. **C**) Spontaneous and MSP-induced phosphorylation of RON, RON160, and RON^E5/6in ^in MDCK cells. Cells (3 × 10^6 ^cells/sample) after 72 h incubation were stimulated at 37°C with or without 2 nM of MSP for 10 min. Cellular proteins (250 μg/sample) were immunoprecipitated with Zt/g4 (1 μg/ml) followed by Western blot analysis using anti-phosphotyrosine mAb PY-100. Membranes were also reprobed with rabbit anti-RON C-terminus antibodies as the loading control. **D**) Effect of MSP on RON, RON160, and RON^E5/6in^-mediated phosphorylation of downstream signaling proteins. Cells were stimulated for 10 min with MSP as described in C. Western blot analysis using antibodies to regular or phosphorylated Erk1/2 and AKT were carried out as previously described [29]. Data shown here are from one of three experiments with similar results.

Since the deletion of the first IPT unit leads to oncogenic conversion [13], we wanted to know if insertion in the same domain has a similar effect. To this end, the cDNA encoding the RON^E5/6in ^was constructed by replacing a fragment in wild-type RON cDNA with the cloned Fgm-III and then stably transfected into MDCK cells. Results from Western blot analysis showed that both RON160 and RON^E5/6in ^were processed from single-chain precursor into mature α/β heterodimer (as evident by the presence of the β-chain) (Figure 2A). These indicate that deletion or insertion does not affect the exposure of α/β chain cleavage site (Lys^304^-Arg-Arg-Arg) on the surface of RON for enzymatic conversion. Interestingly, a protein with molecular mass of 110 kDa was observed in M-RON^E5/6in ^cells, which is not observed in RON or RON160 expressing cells (this variant RONp110 will be described later in detail). This suggests that the processing of RON^E5/6in ^differs from RON160. Immunofluorescent analysis showed that both RON160 and RON^E5/6in ^are expressed on the cell surface (Figure 2B), suggesting that synthesized receptors were transported from cytoplasm to cell surface. Analysis of protein phosphorylation revealed that RON160 is constitutively active with high levels of tyrosine phosphorylation. MSP stimulation only marginally enhanced its phosphorylation status (Figure 2C). In contrast, RON^E5/6in ^was not phosphorylated in unstimulated cells. MSP stimulation was required for its phosphorylation (Figure 2C). These results indicate that deletion in the first IPT unit causes spontaneous activation. However, the insertion does not transform the protein into a constitutively active variant. At intracellular signaling, RON^E5/6in^-mediated activation of Erk1/2 and AKT relied on MSP stimulation (Figure 2D). In contrast, Erk1/2 and AKT were constitutively phosphorylated in M-RON160 cells. MSP only slightly enhanced RON160-mediated phosphorylation of Erk1/2 and AKT. Taken together, these results demonstrate that insertion and deletion in the first IPT unit do not affect transportation, maturation, and cell surface expression of RON160 and RON^E5/6n^. However, the deletion resulted in constitutive activation of RON160. In contrast, the insertion failed to convert RON^E5/6in ^into a constitutively active variant.

### Variant RONp110 is generated from RON^E5/6in ^and RON but not from RON160 in response to cell-derived proteases

As shown in Figure 2A, the expression pattern of RON^E5/6in ^differs from wild-type RON and RON160 with an additional RON variant (RONp110). Analysis by protein micro-sequencing revealed that RONp110 is a proteolytic cleaved and truncated protein missing the majority of the extracellular sequence (Figure 1A and 1B). The N-terminal first amino acid was Lys^632^, which is in the middle of the first IPT unit coded by exon 6. Consistent with these analyses, we detected a soluble RON isoform with molecular mass of ~80 kDa (designated as RON^Er80^) from culture fluids under non-reduced conditions. This protein was not observed in cells expressing RON^E5/6in ^(data not shown). These results indicate that RON^E5/6in ^is proteolytically processed to form RONp110 and RON^Er80^. Analysis of amino acids adjacent to Lys^632 ^showed that the sequence Val-Pro-Arg-**Lys**^632^-Asp-Phe-Val is highly susceptible to digestion by trypsin-like serine proteases [35]. This indicates that insertion in the first IPT unit facilitates the exposure of this particular sequence for potential digestion by trypsin-like serine proteases. In contrast, deletion of the first IPT unit eliminates this sequence. Therefore, RON160 is resistant to trypsin-like serine proteases.

**Figure 3:**
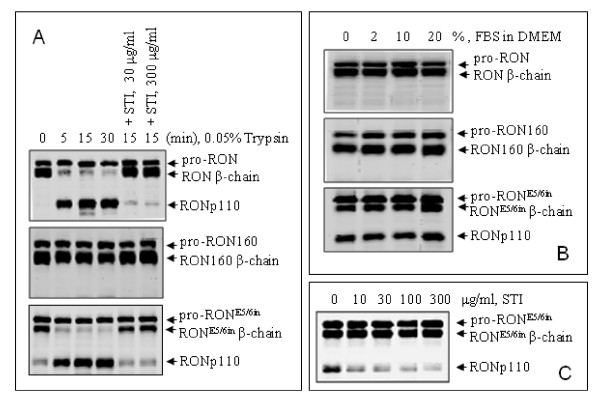
**Generation of RONp110 from RON**^**E5/6in **^**but not from RON160 in response to trypsin or cell-derived trypsin-like proteases**. **A) **M-RON, M-RON160 and M-RON^E5/6in ^cells (2 × 10^6 ^cells per sample) were incubated for 24 h and then treated with 0.05% of trypsin in the presence or absence of STI in DMEM at 37°C for 5, 15, and 30 min. Proteins (50 μg per sample) from cell lysates were analyzed by Western blot analysis using rabbit anti-RON antibody. **B**) Cells were incubated for 72 h and then treated at 37°C with different amounts of FBS in DMEM for 15 min. Western blot analysis was performed as described in B. **C**) Effect of STI on RONp110 formation mediated by cell-associated proteases. M-RON^E5/6in ^cells in serum-free DMEM were treated at 37°C with different amounts of STI for 48 h. Levels of RONp110 in cell lysates were determined by Western blot analysis as detailed in C. Results shown here were from one of three experiments with similar results.

To confirm this, trypsin was used to treat M-RON160 and M-RON^E5/6in ^cells followed by Western blot analysis. M-RON cells were used as the control. Results in Figure 3A showed that RON expression in MDCK cells did not result in RONp110 formation. RONp110 was only produced when M-RON cells were treated with trypsin in a time-dependent manner. In contrast, RONp110 existed in M-RON^E5/6in ^cells in the absence of trypsin. The amounts of RONp110 were dramatically increased after trypsin treatment. As expected, RONp110 was not produced from RON160 when M-RON160 cells were treated with trypsin. Results in Figure 3A further confirmed that treatment of cells with soybean trypsin inhibitor (STI) blocks trypsin activity, which inhibits RONp110 generation. Thus, RONp110 generation is mediated by enzymatic cleavage at the digestive site of Val-Pro-Arg-**Lys**^632^-Asp-Phe-Val. Expression of RON^E5/6in ^spontaneously causes RONp110 formation. Both RON and RON^E5/6in ^have the potential to produce RONp110 after exogenous trypsin treatment.

To determine the source of trypsin-like proteases, M-RON160, and M-RON^E5/6in ^cells were incubated for 72 h in serum-free or FBS-containing medium. M-RON cells were used as the control. Results from Western blot analysis showed that culture of M-RON cells with increased amounts of FBS does not result in any RONp110 formation, indicating that RONp110 is not produced by M-RON cells under regular culture conditions containing FBS (Figure 3B). Similarly, RONp110 was not generated from M-RON160 cells in the presence or absence of serum. In contrast, RONp110 was produced in M-RON^E5/6in ^cells cultured with serum-free medium (Figure 3C). Addition of serum did not further increase RONp110 production by M-RON^E5/6in ^cells. These results suggest that FBS is not the source for trypsin-like enzymes. It is very likely that cell-associated proteases are responsible for the generation of RONp110 in M-RON^E5/6in ^cells.

To determine if cell-derived proteases are sensitive to inhibition by trypsin inhibitors, M-RON^E5/6in ^cells in serum-free medium were treated with different amounts of STI. Results in Figure 3C showed that STI inhibits RONp110 formation in a dose-dependent manner, suggesting that although the nature of the enzyme is unknown, cell-associated trypsin-like protease(s) is responsible for the conversion of RON^E5/6in ^into RONp110.

### Cytoplasmic pro-RON160 and pro-RON^E5/6in ^are differentially converted into α/β mature protein

**Figure 4:**
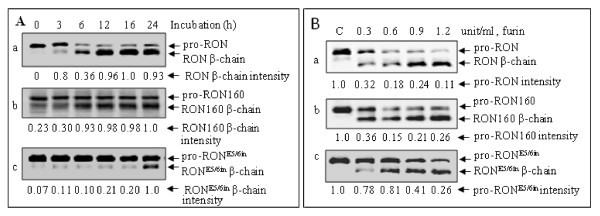
**Differential conversions of pro-RON160 and pro-RON**^**E5/6in **^**expressed in MDCK cells**. **A**) Time-dependent conversion of pro-RON160 and pro-RON^E5/6in ^in MDCK cells. Individual cell lines (2 × 10^6 ^cells/sample) were treated with 0.05% trypsin for 30 min to eliminate mature β-chain and then incubated with DMEM containing 10% FBS for variable hours. Cell proteins were subjected to Western blot analysis as described above Figure 3. **B**) Proteolytic effect of furin on pro-RON160 and pro-RON^E5/6in^. Purified pro-RON, RON160 and RONE^5/6in ^in DMEM were incubated at 37°C with different amounts of furin for 5 h as previously described [37]. The samples were then subjected to Western blot analysis as described above. Protein intensity was determined by VersaDoc Imagining software as previously described [28].

Proteolytic conversion of pro-RON into two-chain mature RON is required for expression on the cell surface and for interaction with MSP [8,36]. By analyzing the levels of precursor and β-chain, the conversion process can be determined. Results in Figure 4A showed the different patterns of proteolytic conversion of pro-RON160 and pro-RON^E5/6in ^in MDCK cells. Using β-chain as an indicator, conversion of pro-RON was seen as early as 3 h, reached maximal level at about 12 h, and then stabilized thereafter. Proteolytic cleavage of pro-RON160 was processed in a manner similar to pro-RON. The mature RON160 β-chain was observed after initiation of cell labeling. Saturated levels of RON160 β-chain were seen around 12 h and maintained thereafter. In contrast, pro-RON^E5/6in ^conversion was significantly delayed in comparison with pro-RON and pro-RON160. Although trace amounts of converted products were observed in the early stages of incubation, significant amounts of RON^E5/6in ^β-chain were detected only after cells were incubated for 24 h. Stabilized RON^E5/6in ^β-chain was seen mainly at 72 h of incubation (data not shown).

Proteolytic conversion of the MET precursor is mediated by members of the subtilisin-like proprotein convertase family such as furin, which has the preferred Arg-X-Lys/Arg-Arg sequence as the cleavage site [37,38]. We tested if delayed maturation is caused by insensitivity of pro-RON^E/56in ^to furin-mediated cleavage. After purification by Zt/g4 immunoprecipitation, individual samples of pro-RON, pro-RON160, and pro-RON^E5/6in ^were treated with various amounts of recombinant furin at 37°C and the conversion was evaluated by Western blot analysis. As shown in Figure 4B, pro-RON and pro-RON160 were correctly cleaved by furin in a dose-dependent manner. In contrast, pro-RON^E5/6in ^was relatively insensitive to furin-mediated cleavage. When treated with 0.6 unit/ml of furin, only small amounts of pro-RON^E5/6in ^were converted to the mature β-chain. Thus, pro-RON^E5/6in ^is relatively insensitive to enzymatic cleavage by protein convertase furin.

### Down-regulation of RON^E5/6in ^but not RON160 is significantly accelerated upon anti-RON mAb engagement

**Figure 5:**
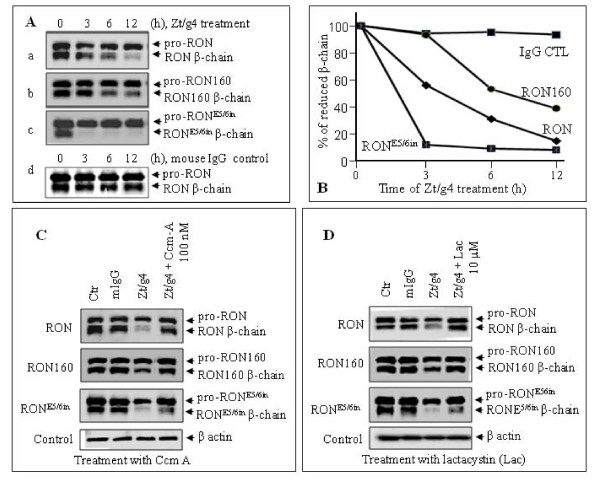
**Accelerated down-regulation of RON**^**E5/6in **^**but not RON160 upon anti-RON mAb treatment**: **A) **Zt/g4-induced receptor down regulation. M-RON, M-RON160 and M-RON^E5/6in ^cells (2 × 10^6 ^cells per sample) were treated at 37°C with 10 μg/ml of Zt/g4 for various intervals. Normal mouse IgG was used as the negative control. Cellular proteins (50 μg/ml per sample) from cell lysates were subjected to Western blot analysis using rabbit anti-RON C-terminus antibody. **B**) Intensity of individual protein bands from different groups in **A **were compared and plotted against various time points after Zt/g4 treatment. **C**) Preventive effect of Ccm A on Zt/g4-induced receptor down-regulation. Cells were treated for 12 h as described above. Ccm A was added after initiation of Zt/g4 treatment followed by Western blot analysis. **D**) Preventive effect of lactacystin on Zt/g4-inuced receptor down-regulation. Cells were treated for 12 h as described above. Lactacystin was added after initiation of Zt/g4 treatment followed by Western blot analysis. Data shown here are from one of three experiments with similar results.

The differences between RON160 and RON^E5/6in ^prompted us to study if RON^E5/6in ^differs from RON160 in receptor internalization process. Anti-RON mAb Zt/g4-induced RON internalization and degradation [39] was used as the model. Results in Figure 5A show a time-dependent internalization of RON160 and RON^E5/6in ^after Zt/g4 treatment. Quantitative values are presented in Figure 5B. Zt/g4-induced RON internalization was used as the control. After Zt/g4 treatment for 12 h, more than 80% of mature RON (evident by levels of the RON β-chain) was internalized followed by degradation. Zt/g4-induced RON160 internalization was significantly delayed than that of wt-RON. Only 60% of mature RON160 was down-regulated 12 h after Zt/g4 treatment. In contrast, the down-regulation of RON^E5/6in ^was significantly accelerated. After Zt/g4 treatment for 3 h, almost all mature RON^E5/6in ^was internalized followed by degradation. These results suggest that RON^E5/6in ^internalization and down-regulation is significantly accelerated upon Zt/g4 engagement. In contrast, RON160 displayed relative resistance to Zt/g4-induced internalization and degradation.

Chemical inhibitors, concanamycin A (Ccm-A) and lactacystin that specifically inhibit lysosome and proteoasome-mediated protein degradation, respectively [40,41], were used to determine how internalized proteins were degraded. Results in Figure 5C show the preventive effect of Ccm-A on lysosome-mediated degradation of RON, RON160, and RON^E5/6in ^in MDCK cells. Although Ccm-A almost completely prevented Zt/g4-induced down-regulation of RON and RON160, it showed only a moderate effect on prevention of RON^E/5/6in ^degradation. Similar results were also observed when proteoasome inhibitor lactacystin was used (Figure 5D). In this case, lactacystin almost completely prevented RON and RON160 degradation. However, degradation of RON^E5/6in ^was only partially prevented by lactacystin. These results suggest that inhibition of lysosome or proteoasome-mediated degradation prevents Zt/g4-induced RON and RON160 down-regulation. However, Ccm-A or lactacystin alone only partially prevents Zt/g4-induced degradation of RON^E5/6in^.

### Functional differences between RON160 and RON^E5/6in ^in regulating tumorigenic activities

**Figure 6:**
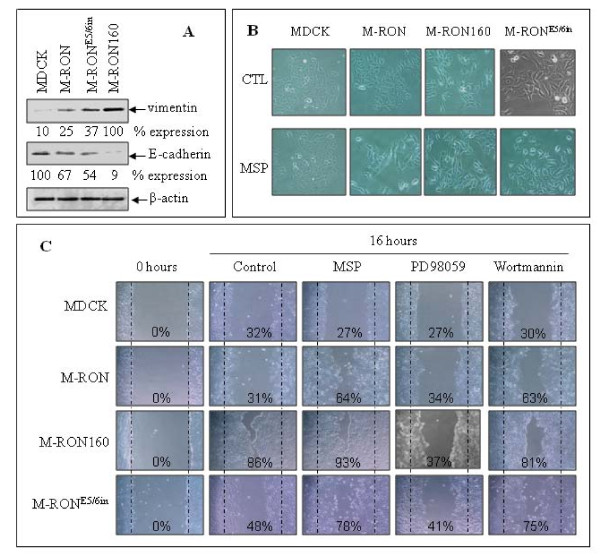
**Regulatory effect of RON160 and RON**^**E5/6in **^**on EMT-like activities in MDCK cells**: **A) **Effect of RON, RON160, and RON^E5/6in ^on epithelial/mesenchymal protein expression. Proteins (50 μg per sample) from cell lysates prepared after 72 h incubation were subjected to Western blot analysis using antibodies specific to vimentin and E-cadherin, respectively. Actin was used as the loading control. **B**) Effect of RON, RON160 and RON^E5/6in ^on cell morphological changes. MDCK, M-RON, M-RON160 and M-RON^E5/6in ^cells were cultured for 24 h and then stimulated with 2 nM of MSP for 48 h. Cell morphological changes were observed under Olympus Inverted microscope and photographed. **C**) Effect of RON, RON160 and RON^E5/6in ^on spontaneous or MSP-induced MDCK cell migration. Cell monolayer was wounded as previously described [29] and stimulated with or without 2 nM of MSP for 16 h. Chemical inhibitors such as PD98059 (10 nM, specific to MAP kinase) and wortmannin (50 μg/ml, specific to PI-3 kinase) were added simultaneously. The wounded area covered by migrated cells was measured and shown as % of the covered space. Data shown here are from one of three experiments with similar results.

Overexpression of RON and RON160 in epithelial cells results in EMT-like phenotype [15,16], which is characterized by reduced E-cadherin expression (epithelial marker) and appearance of vimentin (mesenchymal protein) (Figure 6A). Such changes were also observed in M-RON^E5/6in ^cells; in which vimentin is expressed and levels of E-cadherin are reduced, although the levels of expression were not as obvious as RON160. Morphological analysis of cell shape also showed that RON^E5/6in ^expression moderately causes cell morphological change (Figure 6B). In contrast, RON160 expression significantly altered cell morphologies. Scatter-like activities mediated by RON160 upon MSP stimulation were more significant in M-RON160 than in M-RON^E5/6in ^cells.

Results from analysis of cell migration showed that expression of RON^E5/6in ^moderately increases spontaneous migration of MDCK cells (from 0% to 48%). The migration was further enhanced by MSP stimulation (from 53% to 78%). In contrast, RON160 expression significantly increases spontaneous migration (from 0% to 86%) (Figure 6C). MSP stimulation also slightly enhanced this activity (from 86% to 93%). Experiments using MAP kinase (PD98059) or PI-3 kinase (wortmannin) inhibitors further showed that spontaneous or MSP-induced migration is preventable by addition of PD98058 in all cell lines tested. In contrast, the effect of wortmannin was minimal. These results demonstrate that RON160 is a much stronger molecule than RON^E5/6in ^in induction of EMT and cell migration.

**Figure 7:**
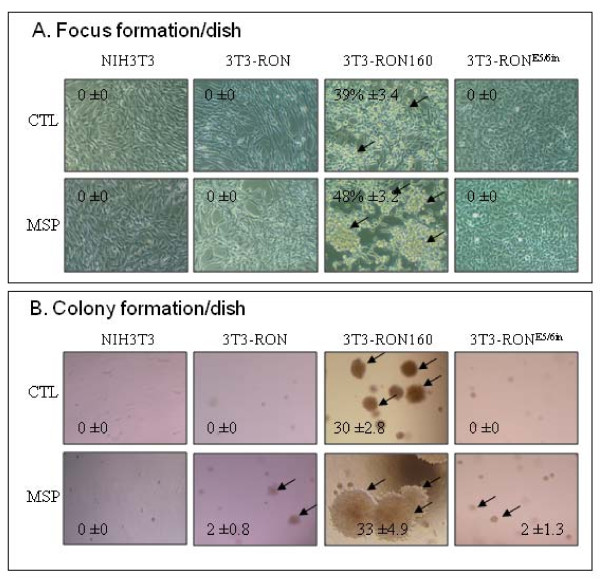
**Effect of RON, RON160, and RONE5/6in on focus formation and anchorage independent growth**: **A**) Focus formation by transformed NIH3T3 cells. NIH3T3 cells (1 × 10^5 ^cells per dish) were transiently transfected with 3 μg of individual pcDNA3 expression vector containing RON, RON160 or RON^E5/6in ^cDNA as previously described [13]. After incubation for 48 h, cells in 2% FBS-DMEM were stimulated with or without 2 nM of MSP for additional 8 days. Focus formation was observed, photographed, and counted from individual groups. **B**) Colony formation in soft agar by transfected NIH3T3 cells. The soft agar assays were performed as previously described [13]. NIH3T3, 3T3-RON, 3T3-RON160, and 3T3-RON^E5/6in ^cells (1000 cells per dish) were seeded in soft agar with or without 2 nM of MSP for 25 days. Cell growth at ≥30 cells per cluster was marked as a positive colony and counted.

We further studied the effect of RON160 and RON^E5/6in ^on induction of focus formation and anchorage-independent growth in soft agar using NIH 3T3 cells as the model. Results in Figure 7A showed that transient expression of RON^E5/6in ^does not cause focus formation by NIH-3T3 cells. MSP stimulation also failed to induce focus formation in RON^E5/6in ^expressing cells. These results were in line with cells expressing wild-type RON, which is known as a non-transforming agent [42]. In contrast, RON160 expression resulted in numerous large-sized foci in transfected NIH3T3 cells. Although MSP stimulation only moderately increased the number of foci, it dramatically enlarged the size of these foci.

Consistent with focus formation studies, results from the soft agar experiments showed that RON^E5/6in ^expression does not lead to colony formation in soft agar. Addition of MSP only marginally stimulated a few small-sized colonies grown in soft agar. In contrast, RON160 expression resulted in numerous colony growths in soft agar. This effect was further enhanced after MSP is added to cell culture. Moreover, the size of individual colonies was much bigger than those in unstimulated 3T3-RON160 cells. Thus, like wild-type RON, RON^E5/6in ^is not a transforming agent. Its expression is not sufficient to cause focus and colony formation. In contrast, RON160 is a strong transforming agent, which can be verified in both focus and colony formation assays.

## Discussion

The findings in this study demonstrate that alterations in the first IPT unit in the RON extracellular sequence results in two novel variants with different biological profiles. Structurally, the IPT units consist of 80 to 100 amino acids and are featured by immunoglobulin-like fold [25,43]. The IPT units are also found in certain transcription factors such as NF-κB and c-Jun, where it is involved in protein-protein and/or protein-DNA interaction [44]. The significance of the IPT units in MET and RON has recently been discovered and emphasized. The deletion of the first IPT unit in the RON extracellular sequences converts wild-type RON into oncogenic agent RON160 [13], although the underlying mechanisms are unknown. In MET, the fourth IPT unit in the β-chain extracellular sequence harbors a high affinity binding site for ligand HGF/SF [45]. HGF/SF binding to this IPT unit is essential for induction of MET activation [45]. Clearly, these findings illustrate the importance of the IPT units in MET/RON-mediated signaling cascades and tumorigenic activities. The data from our current studies demonstrate that deletion or insertion in the RON first IPT unit exerts different consequences. Although deletion of the first IPT unit leads to oncogenic conversion, insertion of 20 amino acids in the same unit is not sufficient to transform the RON protein into an oncogenic agent. However, insertion has important impact on biochemical properties of RON. We show that proteolytic conversion of pro-RON^E5/6in ^into the two-chain mature protein by convertase furin is significantly delayed upon precursor synthesis. RON^E5/6in ^is also highly susceptible to cell-associated serine proteases, which act on a short sequence leading to generation of another variant RONp110. Moreover, RON^E5/6in ^is internalized in an accelerated manner upon anti-RON mAb engagement. Thus, alterations in the first IPT unit differentially regulate RON-mediated activity with different biochemical properties. In addition, generation of RON160 and RON^E5/6in ^provides an opportunity to understand the roles of IPT units in regulating RON activation and activity, which could aid to develop therapeutic agents for inhibition of RON-mediated tumorigenic signaling.

Overexpression of RON in cancerous tissues is often accompanied with the generation of aberrant mRNA transcripts and their corresponding variants [13,14]. This has been considered as a mechanism by which RON displays its protein diversity and regulates epithelial homeostasis and malignant transformation [7]. A survey by PCR of primary colon, lung, breast, and brain tumor samples has revealed that aberrant mRNA transcripts encoding for known and unknown variants such as RON165, RON160, and RON155 were wildly produced with relatively high frequencies in colon, breast, lung and other types of cancers [46]. These variants are mainly generated by aberrant mRNA splicing processes that delete exon 11 (RON165), exons 5 and 6 (RON160), and exons 5, 6 and 11 (RON155) [7,46]. It needs to be emphasized that exon 11 encodes 49 amino acids belonging to the fourth IPT unit in the RON β-chain extracellular sequences [25], which is required for pro-RON maturation and cell surface localization [28]. Results in current studies demonstrate that alterations in the first IPT unit in the RON protein are not a rare occurrence. Among 12 cancer cell lines analyzed, abnormality in the first IPT unit was observed in 5 cell lines originating from colon, breast and pancreatic tumors. These results are consistent with those from analysis of primary tumor samples [14,46]. As reported, deletion of exons 5 and 6 were observed in more than 50% of primary colon and 90% of brain tumor samples but not in any normal tissues [14,46]. Further analysis of insertions between exons 5 and 6 using clinical tumor samples would be very informative. Although the underlying mechanisms of variant generation are currently unknown, it is known that aberrant splicing and intron retention in receptor tyrosine kinases occur commonly in cancer cells [47,48]. Considering the oncogenicity is of RON160 *in vivo*, such alterations with high frequencies should have pathogenic significance in relevance to tumor progression and malignant phenotypes.

From the viewpoint of disrupting the first IPT unit, it was a surprise that RON^E5/6in ^differs significantly from RON160 in terms of their biochemical and biological properties (Table 2). First, RON160 and RON^E5/6in ^both are cleaved from precursor into respective mature forms but the kinetics of their processing is different (Figure 4). Pro-RON160 is cleaved at a rate similar to that of pro-RON. In contrast, RON^E5/6in ^is matured at relatively late stages. This is probably due to relative insensitivity of RON^E5/6in ^to convertase furin-mediated proteolytic cleavage. Such insensitivity could be due to sequence alterations in the insertion. Site-directed mutagenesis may verify if this is the case. Another possibility is that insertion-induced conformational change may affect the access of furin to the cleavage site located at α/β chain junction. Second, under regular culture conditions containing FBS, RON^E5/6in ^is the major source for generation of RONp110, although wild-type RON can also be truncated by exogenous trypsin to form RONp110. This suggests that the insertion causes the digestion site (Val^629^-Pro-Arg-Lys-Asp-Phe) in the first IPT unit more accessible to cell-associated trypsin-like serine proteases. As a post-translational truncated product, RONp110 misses the majority of the extracellular domains including sema, PSI, and a large portion of the first IPT. MSP stimulation hardly induced its phosphorylation (Figure 1B and 2C), which suggests that MSP may not bind to RONp110. As expected, enzymatic digestion of RON or RON^E56in ^by cell-derived trypsin-like proteases also produce a soluble ~80 kDa RON extracellular isoform (RON^Er80^) comprising the entire 35 kDa α-chain and a ~45 kDa partial extracellular β-chain. The isoform is similar to a previously reported RONΔ85 [24]. RONΔ85 is a soluble truncated RON variant produced by a mRNA transcript from a breast cancer cell line with insertion of 49 nucleotides between exons 5 and 6 [24]. RONΔ85 has the inhibitory effect on MSP-induced RON signaling events [24]. Considering their structural similarities, it is reasoned that the RON^Er80 ^may have the ability to regulate MSP-induced RON-mediated activities. Currently, the role of RONp110 is unknown. Interestingly, a similar variant of MET lacking the ectodomain but retaining the transmembrane and intracellular domains has been discovered in several cancer samples [49]. This protein resides on the cell surface and displays transforming, invasive, and tumorigenic activities [49]. Third, deletion of the first IPT unit results in constitutive tyrosine phosphorylation [13]. In contrast, insertion does not convert RON^E5/6in ^into constitutive phosphorylation. RON^E5/6in ^remains inactive and requires MSP stimulation for phosphorylation and activation of downstream signaling molecules such as Erk1/2 and AKT (Figure 2C). Previous studies have shown that deletion of first IPT unit results in imbalance of cysteine residues in the extracellular sequences, a possible reason for spontaneous dimerization leading to constitutive phosphorylation [13]. Interestingly, a cysteine residue was seen in the inserted 20 amino acids in the RON^E5/6in ^molecule, which also causes an imbalance in the number of cysteine residues in the extracellular sequence. However, such addition does not seem to affect the extracellular conformation of RON^E5/6in^. Thus, additional mechanism(s) is probably involved in constitutive activation of RON160. Fourth, RON160 is relatively resistant to anti-RON mAb-induced internalization and degradation. In contrast, RON^E5/6in ^is highly susceptible to Zt/g4-mediated degradation (Figure 5). At present, we do not know mechanism(s) responsible for such an accelerated process. However, this is important for RON160 to sustain its intracellular oncogenic signaling. As reported previously, oncogenic RON variants created by mutations in the kinase domain are highly resistant to ligand-induced internalization [50]. We have previously found that anti-RON mAb-induced down-regulation attenuates RON-mediated tumorigenic signaling and motile-invasive activities in colon cancer cells [39]. Thus, insertion in the first IPT unit, through an unknown mechanism, accelerates antibody-induced RON^E5/6in ^internalization and degradation. Finally, insertion and deletion in the first IPT showed differential effects on cellular activities. From functional analysis, RON^E5/6in ^mediated EMT-like activities are similar to those mediated by wild-type RON. However, as judged by levels of vimentin and E-cadherin, changes in cell morphologies, and cell motility, RON160 is much more potent than RON^E5/6in ^in mediating these tumorigenic activities. Analysis of cell transforming and anchorage-independent activities further demonstrate that insertion in the first IPT unit does not convert wild-type RON into a transforming agent. It is the deletion that renders RON160 as the transforming variant. As evident by *in vitro *transforming assays, the number of foci mediated by RON160 was significantly higher than that in RON^E5/6in ^expressed NIH3T3 cells. Anchorage-independent growth by colonies in soft agar was also observed only in RON160 expressing NIH3T3 cells. Thus, alterations in the first IPT unit, either by insertion or deletion, result in two RON variants with distinct structural and cellular activities.

**Table 2:** Biochemical and Biological Differences between RON160 and RON^E5/6in^

Features	Similarity and Difference*:		
	
	RON	RON160	**RON**^**E5/6in**^
mRNA in tumor cell lines	11 out of 12 in CC, BC, PC lines	4 out of 12 in CC, BC lines	4 out of 12 in CC, BC, PC lines.
First IPT unit	wild-type	exon deletion	intron retention
Protein location	cell surface	cell surface	cell surface
Activation	MSP required	constitutively active	MSP required
Response to MSP	strong	moderate	strong
Trypsin digestion	sensitive	no effect	sensitive
Generation of RONp110	upon trypsin treatment	no effect	spontaneous and trypsin treatment
Furin treatment	sensitive	sensitive	less sensitive
Intracellular degradation	sensitive	less-sensitive	highly sensitive
Induction of EMT	moderate effect	strong effect	moderate effect
Cell migration	moderate effect	highly effect	moderate effect
Transforming activity	no effect	strong effect	no effect
Colony formation	no effect	strong effect	no effect

*CC, colon cancer; BC, breast cancer; and PC, pancreatic cancer.

## Competing interests

The authors declare that they have no competing interests.

## Authors' contributions

QM carried out most biochemical and biological studies. KZ did RT-PCR and cDNA cloning studies. SG performed internalization/degradation experiments. YQZ and MHW participated in the design of the study and draft the manuscript. All authors have read and approved the final manuscript.
